# Blood Magnesium Level and Selected Oxidative Stress Indices in Lead-Exposed Workers

**DOI:** 10.1007/s12011-020-02168-x

**Published:** 2020-05-06

**Authors:** Magdalena Wyparło-Wszelaki, Marta Wąsik, Anna Machoń-Grecka, Aleksandra Kasperczyk, Francesco Bellanti, Sławomir Kasperczyk, Michał Dobrakowski

**Affiliations:** 1 Department of Biochemistry, Faculty of Medical Sciences in Zabrze, Medical University of Silesia, ul. Jordana 19, 41-808 Zabrze, Poland; 2 Department of Medical and Surgical Sciences, University of Foggia, viale Pinto, 1, 71122 Foggia, Italy

**Keywords:** Magnesium, Oxidative stress, Lead exposure

## Abstract

Occupational exposure to lead is one of the important hazards to human global population. Lead interferes with divalent cations, such as calcium, magnesium, and iron. Magnesium is the fourth most common mineral in the human body and a cofactor in more than 325 enzymes. There are many disorders associated with magnesium deficiency. It has been postulated that hypomagnesemia promotes oxidative stress. Study population included 232 male employees of lead-zinc works and was divided into two sub-groups based on the median of magnesium serum level: low magnesium level (L-Mg) group and high magnesium level (H-Mg) group. Magnesium level was significantly higher in the H-Mg group than in the L-Mg group due to the study design. The level of zinc protoporphyrin was significantly higher in the L-Mg group than in the H-Mg group by 13%, while the blood lead levels were similar in the examined groups. The serum level of MDA was significantly higher in the L-Mg group than in the H-Mg group by 12%, while the serum levels of thiol groups, TAC, and bilirubin were significantly lower in that group by 6%, 3%, and 27%, respectively. Similarly, the erythrocyte SOD activity was lower in the L-Mg group than in the H-Mg group by 5%. Low serum magnesium levels contribute to lead-induced oxidative stress, result in unfavorable modification of antioxidant system function, and promote lead-induced impairment of heme synthesis. Obtained results indicate that prevention of hypomagnesemia should be regarded as an important step in ensuring adequate prophylaxis of chronic lead poisoning.

## Introduction

Heavy metals have become a global health problem. One of them is lead that is toxic to humans and animals. Lead is widespread in the environment, including water, soil, and dust. Lead can be also present in manufactured products. Therefore, environmental and occupational exposure to lead is one of the important hazards to human global population [1]. Lead together with mercury and cadmium are classified as the most relevant toxic substances in general and are included in the top 10 chemicals of major public health concern according to the World Health Organization (WHO) [2].

The gastrointestinal and respiratory tracts are the main routes of lead absorption. Lead affects all body systems. The most susceptible to lead toxic effects are erythropoiesis, kidney function, and the central nervous system function [2]. Lead affects adversely also the cardiovascular system and increases the incidence of hypertension, coronary artery disease, left ventricular hypertrophy, alterations in cardiac rhythm, and peripheral artery disease [3].

There are many possible mechanisms of lead toxicity. Thanks to a strong electron sharing property, lead forms covalent bonds with the sulfhydryl groups of proteins, making the enzymes most susceptible targets for lead, including antioxidant enzymes. Lead neutralizes also glutathione (GSH) that serves as the major thiol antioxidant. These interferences result in an imbalance between production and neutralization of reactive oxygen species (ROS) due to lead action [4]. Besides, lead has pro-inflammatory properties associated with its influence on the cytokine metabolism, the expression and activity of enzymes involved in the inflammation, and the levels of acute-phase proteins [5]. Lead also interferes with divalent cations, such as calcium, magnesium, and iron. By their substitution, lead can affect various fundamental biological processes [3].

Magnesium is the fourth most common mineral in the human body and a cofactor in more than 325 enzymes. There are many disorders associated with magnesium deficiency, such as hypertension, atherosclerosis, cardiac arrhythmias and infarct, dyslipidemia, insulin resistance, metabolic syndrome, osteoporosis, and neuropsychiatric disorders [6]. It has been postulated that hypomagnesemia promotes oxidative stress. First of all, decreased level of magnesium impairs function of respiratory chain and leads to mitochondrial dysfunction and increased ROS release. Secondly, hypomagnesemia results in increased levels of pro-inflammatory cytokines and neutrophils as well as macrophages activation. These cells act as a source of superoxide anions due to NADPH oxidase activity. Thirdly, decreased ratio of magnesium to calcium is supposed to increase levels of catecholamines and activate renin-angiotensin-aldosterone axis that also induces oxidative stress. Fourthly, magnesium is believed to protect cell membranes from oxidative damage. On the other hand, oxidative stress favors hypomagnesemia [6, 7].

In light of the above-mentioned information, it can be hypothesized that low magnesium levels may enhance lead toxicity in respect of oxidative stress and inflammatory response. In parallel, lead-induced oxidative stress may contribute to hypomagnesemia as a positive feedback mechanism. However, there is limited information in the available literature on relating lead and magnesium [8]. Therefore, we aimed to investigate associations among lead, magnesium, and oxidative stress parameters in chronically lead-exposed workers.

## Materials and Methods

### Study Population

Study protocol was approved by the Bioethics Committee of Medical University of Silesia (Permission No. KNW/022/KB1/108/14).

Examined population included 232 male employees of lead-zinc works in Miasteczko Śląskie, Poland. During prophylactic medical examination, epidemiological data were collected, such as age, duration of employment in lead exposure, smoking habits, and medical history. Besides, body mass and height were measured to calculate body mass index (BMI). Lead exposure was estimated based on blood levels of lead and zinc protoporphyrin (ZPP). Inclusion criteria for the study were as follows: occupational exposure to lead and blood lead level exceeding 25 μg/dl.

The study population was divided into two sub-groups based on the median of magnesium serum level (0.862 mmol/l): low magnesium level group (L-Mg group, *n* = 116) and high magnesium level group (H-Mg group, *n* = 116). Serum magnesium level ranged 0.863–1.543 mmol/l in the H-Mg group and 0.629–0.861 mmol/l in the L-Mg group.

This work was supported by Medical University of Silesia (Grant No. KNW-2-08/N/8/N).

### Laboratory Procedures

Blood samples from each subject were collected from the cubital vein using tubes (Vacuette; Greiner-Bio, Frickenhausen, Germany) coated with K_3_EDTA to obtain whole blood, serum, and erythrocytes.

Assessments of the PbB were performed using graphite furnace atomic absorption spectrometry in an ICE 3400 (Thermo Fisher Scientific, Waltham, MA, USA). The results were expressed as μg/dl.

The blood concentration of zinc protoporphyrin (ZPP) was measured directly using Aviv Biomedical hematofluorometer model 206, using an excitation wavelength of 415 nm and an emission wavelength of 596 nm. The results were expressed as μg ZPP per g of hemoglobin (μg/g Hb).

Assessments of the magnesium levels were performed using graphite furnace atomic absorption spectrometry in an ICE 3400 (Thermo Fisher Scientific, Waltham, MA, USA). The results were expressed as mmol/l.

The analyzer Sysmex K-4500 (GMI, MN, USA) was used to determine parameters of the blood morphology: white blood cell (WBC) count, red blood cell (RBC) count, hemoglobin (HGB) level, hematocrit (HCT), platelet (PLT) count.

Total antioxidant capacity (TAC) was measured in serum according to Erel [9]. Data were expressed as mmol/l. Total oxidant status (TOS) was measured in serum according to Erel [10]. Data were expressed as μmol/l. Oxidative stress index (OSI) was calculated as the percentage ratio of TOS to TAC.

The serum concentration of bilirubin was measured using PerkinElmer biochemical analyzer according to the manufacturer’s instructions. Bilirubin concentration was expressed as μmol/l.

The method of Arab and Steghens [11] was used to measure the concentrations of lipid hydroperoxides (LPH) in serum. The results were shown in μmol/l.

The level of malondialdehyde (MDA), a product of lipid peroxidation, was measured fluorometrically as a 2-thiobarbituric acid-reactive substance (TBARS) in serum according to Ohkawa, Ohishi, and Yagi [12] with modifications. Samples were mixed with 8.1% sodium dodecyl sulfate, 20% acetic acid, and 0.8% 2-thiobarbituric acid. After vortexing, samples were incubated for 1 h in 95 °C and butanol-pyridine 15:1 (v/v) was added. The mixture was shaken for 10 min and then centrifuged. The butanol-pyridine layer was measured fluorometrically at 515-nm and 522-nm excitation (PerkinElmer, USA). TBARS values were expressed as malondialdehyde (MDA) equivalents. Tetraethoxypropane was used as the standard. Concentrations were given in μmol/l (serum) and in nmol/g Hb (erythrocytes).

Concentration of lipofuscin (LPS) was measured according to the method of Tsuchida et al. [13]; results were shown in RU/l (relative unit, RU) and RU/g Hb (erythrocytes).

Protein sulfhydryl groups (PSH) concentration was determined as described by Koster, Biemond, and Swaak [14] using DTNB, which undergoes reduction by compounds containing sulfhydryl groups, yielding the yellow anion derivative, 5-thio-2-nitrobenzoate, which absorbs at a wavelength of 412 nm using an automated analyzer PerkinElmer. The results were shown in μmol/g protein and μmol/l.

Ceruloplasmin (CER) concentration in serum was determined as described by Richterich [15]. The results were shown in mg/dl.

The method of Oyanagui [16] was used to measure the activity of SOD in serum and erythrocytes. Activities of superoxide dismutase (SOD) were expressed in NU/ml (serum) and NU/mg Hb (erythrocytes). Catalase (CAT) activity in erythrocytes was measured by the method of Johansson and Håkan Borg [17] using an automated analyzer PerkinElmer. The activity of CAT was expressed as kIU/g Hb. The activity of glutathione reductase (GR) in erythrocytes was measured according to Richterich [15] using an automated analyzer PerkinElmer. The activity was expressed as μmoles of NADPH utilized per minute per g hemoglobin in erythrocytes (IU/g Hb). The activity of glutathione S-transferase (GST) in erythrocytes was measured according to the kinetic method of Habig and Jakoby [18] using an automated analyzer PerkinElmer. The activity of GST was expressed as μmoles of thioether produced per minute per g hemoglobin in erythrocytes (mIU/g Hb). Glutathione peroxidase (GPx) activity in erythrocytes was measured by the kinetic method of Paglia and Valentine [19]. The activity of GPx was expressed as micromoles of NADPH oxidized per minute per g hemoglobin in erythrocytes (IU/g Hb).

### Statistical Analysis

Statistical analysis was performed using Statistica 10.0 PL software. Results were reported as mean ± standard deviation (SD) for normal distribution. Shapiro-Wilk’s test was used to verify normality, and Levene’s test was used to verify homogeneity of variances. Statistical comparisons were made using a *t* test, a *t* test with separate variance estimates, a Mann-Whitney *U* test, or a chi-squared test. The Spearman non-parametric correlation was calculated. Additionally, regression analysis was performed. A *p* value < 0.05 was considered statistically significant.

## Results

The mean values of age, employment duration, height, weight, BMI, and smoking habits did not differ between the examined groups (Table 1). The percentages of participants diagnosed with diabetes, arterial hypertension, and coronary artery disease were also not different in the examined groups (Table 1).

**Table 1:** Epidemiologic data and percentages of chronic diseases in low magnesium level group (L-Mg group) and high magnesium level group (H-Mg group).

	L-Mg group		H-Mg group		*p*
	*n*=116		*n*=116		
	Mean	SD	Mean	SD	
Age (year)	40.9	9.77	42.3	9.58	0.285
Employment duration (year)	14.9	8.01	15.4	7.52	0.637
Height (cm)	176	5.92	176	6.76	0.988
Weight (kg)	86.3	12.9	86.1	13.0	0.905
Smoking habits (year)	15.2	8.30	15.0	8.40	0.891
Diabetes	2%		4%		0.353
Coronary artery disease	2%		2%		0.933
Arterial hypertension	15%		13%		0.702

*p* value-*t* test, *p* < 0.05

Magnesium level was significantly higher in the H-Mg group than in the L-Mg group due to the study design. The level of ZPP was significantly higher in the L-Mg group than in the H-Mg group by 13%, while the blood lead levels were similar in the examined groups (Table 2).

**Table 2:** Magnesium level and lead exposure markers in low magnesium level group (L-Mg group) and high magnesium level group (H-Mg group). *PbB* blood lead level, *ZPP* zinc protoporphyrin.

	L-Mg group		H-Mg group		Relative change %	*p*
	Mean	SD	Mean	SD		
Mg (mmol/l)	0.78	0.06	0.97	0.15	24%	<0.001
PbB (μg/dl)	35.3	6.01	34.7	6.13	-2%	0.394
ZPP (μg/g Hb)	5.85	3.36	5.08	2.05	-13%	0.036

*p* value–*t* test, *p* < 0.05

PLT count was significantly lower in the L-Mg group than in the H-Mg group by 5%, whereas the remaining measured parameters of blood morphology were not different between the examined groups (Table 3).

**Table 3:** Blood morphology parameters in low magnesium level group (L-Mg group) and high magnesium level group (H-Mg group). *WBC* white blood cells, *RBC* red blood cells, *HGB* hemoglobin, *HTC* hematocrit, *PLT* platelets.

	L-Mg group		H-Mg group		Relative change %	*p*
	Mean	SD	Mean	SD		
WBC (G/l)	7.32	1.84	7.04	2.10	− 4%	0.290
RBC (T/l)	4.85	0.38	4.89	0.33	1%	0.455
HGB (g/dl)	15.1	0.89	15.2	0.83	1%	0.422
HTC (%)	42.8	2.47	43.1	2.28	1%	0.433
PLT (G/l)	238	50.6	226	53.5	−5%	0.036

*p* value–*t* test, *p* < 0.05

The serum level of MDA was significantly higher in the L-Mg group than in the H-Mg group by 12%, while the serum levels of thiol groups, TAC, and bilirubin were significantly lower in that group by 6%, 3%, and 27%, respectively (Table 4). Similarly, the erythrocyte SOD activity was lower in the L-Mg group than in the H-Mg group by 5% (Table 5).

**Table 4:** Oxidative stress markers in serum in low magnesium level group (L-Mg group) and high magnesium level group (H-Mg group). *TOS* total antioxidant status, *LPH* lipid hydroperoxides, *MDA* malondialdehyde, *LPS* lipofuscin, *OSI* oxidative stress index, *TAC* total antioxidant capacity, *PSH* protein sulfhydryl groups, *CER* ceruloplasmin, *SOD* superoxide dismutase.

	L-Mg group		H-Mg group		Relative change %	*p*
	Mean	SD	Mean	SD		
TOS (μmol/l)	10.3	3.49	10.5	4.92	2%	0.752
LPH (μmol/l)	2.91	1.72	3.17	2.20	9%	0.311
MDA (μmol/l)	3.08	1.37	2.72	1.13	−12%	0.025
LPS (RU/l)	583	101	564	120	− 3%	0.216
OSI (%)	0.99	0.76	0.93	0.53	− 5%	0.555
TAC (mmol/l)	1.13	0.11	1.16	0.12	3%	0.014
Bilirubin (μmol/l)	10.7	3.89	13.6	6.50	27%	0.009
PSH (μmol/g protein)	4.13	0.50	4.40	0.57	6%	< 0.001
CER (mg/dl)	43.5	6.82	42.2	5.98	− 3%	0.130
SOD (NU/ml)	20.6	3.99	19.9	3.54	− 3%	0.174

*p* value—*t* test, *p* < 0.05

**Table 5:** Oxidative stress markers in erythrocytes in low magnesium level group (L-Mg group) and high magnesium level group (H-Mg group). *MDA* malondialdehyde, *LPS* lipofuscin, *GR* glutathione reductase, *GST* glutathione S-transferase, *GPX* glutathione peroxidase, *CAT* catalase, *SOD* superoxide dismutase.

	L-Mg group		H-Mg group		Relative change %	*p*
	mean	SD	mean	SD		
MDA (nmol/g Hb)	220	62.60	229	78.07	4%	0.350
LPS (RU/g Hb)	659	207	638	233	− 3%	0.471
GR (IU/g Hb)	6.84	1.51	7.15	1.41	5%	0.101
GST (mIU/g Hb)	190	75.4	175	72.13	− 8%	0.137
GPX (IU/g Hb)	51.4	13.2	51.2	15.07	0%	0.913
CAT (kIU/g Hb)	451	57.3	463	68.22	3%	0.135
SOD (NU/mg Hb)	187	31.3	197	34.75	5%	0.021

*p* value—*t* test, *p* < 0.05

The correlation analysis showed positive correlations between magnesium level and the levels of TAC (*R* = 0.13, *p* = 0.017), thiol groups (*R* = 0.21, *p* = 0.001), and bilirubin (*R* = 0.18, *p* = 0.024) as well as the activity of SOD (*R* = 0.13, *p* = 0.048). Negative correlations between magnesium level and the levels of ZPP (*R* = − 0.26, *p* = 0.005), MDA (*R* = − 0.16, *p* = 0.017), and PLT count (*R* = − 0.15, *p* = 0.026) were also reported (Table 6).

**Table 6:** Correlations between magnesium level and selected parameters. PbB – blood lead level, *ZPP* zinc protoporphyrin, *MDA* malondialdehyde, *TAC* total antioxidant capacity, *PSH* protein sulfhydryl groups, *SOD* superoxide dismutase, *PLT* platelets.

Correlations between magnesium level and the levels of:	*R*	*p*
PbB (μg/dl)	− 0.14	0.143
ZPP (μg/g Hb)	−.26	0.005
MDA (μmol/l)	−0.16	0.017
TAC (mmol/l)	0.13	0.048
PSH (μmol/g protein)	0.21	0.001
bilirubin (μmol/l)	0.18	0.024
SOD (NU/mg Hb)	0.13	0.048
PLT (G/l)	− 0.15	0.026

*R*-values—Spearman’s rank correlation, *p* < 0.05

The regression analysis (Table 7) showed that the levels/activities of measured parameters in the following order: PSH (*β* = 0.22), ZPP (*β* = − 0.19), MDA (*β* = − 0.17), and SOD (*β* = 0.17) are associated with the blood magnesium level.

**Table 7:** Regression analysis between magnesium level and selected parameters. *ZPP* zinc protoporphyrin, *MDA* malondialdehyde, *PSH* protein sulfhydryl groups, *SOD* superoxide dismutase.

	*R*	*R*^2^	*R*^2^-cor	*p*	− 95% CI	+ 95% CI	*β*
ZPP (μg/g Hb)	0.19	0.036	0.030	0.012	− 6.31	− 0.79	− 0.19
MDA (μmol/l)	0.17	0.028	0.022	0.027	− 2.66	− 0.16	− 0.17
PSH (μmol/g protein)	0.22	0.051	0.045	0.003	0.29	1.35	0.22
SOD (NU/mg Hb)	0.17	0.028	0.022	0.027	4.50	74.38	0.17

*p* < 0.05

## Discussion

There are several epidemiological studies that link blood lead level with the levels of major and minor elements. Most of them was conducted on children. The results are not concordant. Li et al. [20] found a positive correlation between blood levels of lead and magnesium in 2457 children (57.21 ± 35.00 μg/l) aged from 6 to 14 years who were recruited in Nanning, China, while Wu et al. [21] reported a negative correlation between blood levels of lead and magnesium in 3181 children (4.29 ± 2.38 μg/dl) aged from 0 to 14 years who were recruited in Bejing, China. Liu et al. [22] and Zhao et al. [23] found no association between blood levels of lead and magnesium in 1551 children (41.16 ± 16.10 μg/l) aged 1 to 72 months recruited in Nanjing, China, and in 1110 children (49.42 ± 20.16), aged 0 to 6 years recruited in Jinan, China, respectively. Długaszek and Skrzeczanowski [24] showed a positive correlation between hair content of lead and magnesium in boys aged 11 to 15 years. Analogical correlations were found in cartilage and anterior cruciate ligament of the knee joints obtained from 22 women aged 52 to 83 years and 8 men aged 42 to 79 years [25]. In the present study, blood magnesium levels did not correlate with blood lead levels and in the majority of study participants were within the normal range. The discrepancies between above-mentioned results are probably due to many possible factors that may interfere with magnesium metabolism as well as lead absorption, distribution, and excretion, such as type of exposure (environmental vs. occupational), duration of exposure, diet, levels of other elements, and many others.

There are many possible mechanisms of lead toxic action. Interferences between lead and hematopoiesis were investigated in animal and human studies. It has been established that lead downregulates activities of three enzymes involved in the heme biosynthesis pathway, δ-aminolevulinic acid synthetase, δ-aminolevulinic acid dehydratase, and ferrochelatase, in a dose-dependent manner. Consequently, lead poisoning results in anemia due to impaired hemoglobin synthesis. Ferrochelatase is a mitochondrial enzyme that catalyzes the insertion of iron into protoporphyrin. The inhibition of ferrochelatase results in the accumulation of protoporphyrin in erythrocytes. Zinc instead of iron binds to protoporphyin to form zinc protoporphyrin (ZPP) which serves as a marker of lead exposure [4]. In the present study, the levels of lead in the blood were not different between the examined groups; however, the level of ZPP was higher in the L-Mg group than in the H-Mg group. Besides, there was a negative correlation between the levels of ZPP and magnesium. A negative association between ZPP level and magnesium level was also shown in the regression analysis. This observation suggests that low magnesium levels may enhance negative impact of lead on heme biosynthesis. On the other hand, this impact seems to be limited and not clinically significant since we simultaneously did not observe any differences between the examined groups in terms of the values of RBC count, HTC, and HBG level. Similarly, there was no difference between the values of the WBC counts in the examined groups.

By contrast to RBC and WBC counts, PLT count was higher in the L-Mg group compared with the H-Mg group. Additively, we observed a negative correlation between magnesium level and PLT count. Lead may interfere with the blood coagulation through many mechanisms, such as endothelial injury, reduced nitric oxide bioavailability, reduced tissue plasminogen activator level, and an increased production of plasminogen activator inhibitor-1. The associations between blood lead level and PLT count are controversial. Several animal studies have shown a positive association between chronic lead intoxication and PLT count, while several human studies have shown opposite results [26]. In our previous study, increased PLT count was reported in workers exposed to lead for a short period of time [27]. Nevertheless, chronic lead exposure did not result in altered PLT count in another group of workers [28]. Studies indicate also associations between serum magnesium level and platelet function; however, exact mechanisms remain unclear. It has been postulated that magnesium decreases platelet hyperactivity and adhesiveness. Reduced platelet aggregability releases them back to the bloodstream and increases PLT count. Besides, magnesium level has been linked with better bone marrow function, including megakaryocytopoiesis [29]. Consistently, Lu et al. [29] showed an inverse relation between serum magnesium level and thrombocytopenia in 8478 participants of The China Health and Nutrition Survey. Besides, Liu et al. [30] reported higher PLT count in patients without type 2 diabetes and central obesity with higher serum magnesium level than in those with lower serum magnesium level. Such observation was not confirmed in patients with diabetes and central obesity. In light of this information, lower PLT count in the L-Mg group than in the H-Mg group should be expected. Opposite results obtained in the present study may be due to possible additional mechanisms that are triggered by lead.

The ability of lead to induce oxidative stress is well-described. In our previous study, we have shown that chronic exposure to lead induces lipid peroxidation and oxidative DNA damage in a dose-dependent manner and modifies activities of antioxidant enzymes [31]. Magnesium deficiency is also believed to induce oxidative stress. There are several proposed mechanisms explaining negative effects of hypomagnesemia. One of them refers to antioxidant system. It has been reported that low serum magnesium level is accompanied with low glutathione level and decreased activity of antioxidant enzymes, such as superoxide dismutase, catalase, and glutathione peroxidase [7] [32]. Consistently, we reported higher serum MDA level in the L-Mg group compared with the H-Mg group. Elevated level of lipid peroxidation marker may be related to simultaneously observed reduced serum level of sulfhydryl groups that is proportional to glutathione level, a major thiol antioxidant of the human body. Besides, reduced level of sulfhydryl groups should be interpreted as a main cause of reduced TAC level that was also observed. In erythrocytes, the levels of MDA did not differ between the examined groups; however, in the L-Mg group, we observed lower SOD activity than in the H-M group which confirms above-mentioned associations between magnesium and antioxidant enzymes. Differences between the examined groups in terms of the mentioned parameters related to oxidative stress and antioxidant defense are also supported by the regression analysis and correlations. These data confirm a hypothesis that low magnesium levels may enhance lead-induced oxidative stress and aggravate clinical manifestations of lead poisoning. Discussed above associations among lead, magnesium, and PLT count and function as well as mentioned disorders associated with hypomagnesemia, such as hypertension, atherosclerosis, cardiac arrhythmias, and infarct, led us to hypothesize that the coincidence of lead exposure and magnesium deficiency may be especially harmful to the cardiovascular system function.

Associations between lead and non-enzymatic antioxidants are less understood than those with antioxidant enzymes. In our previous studies on chronically and subchronically lead-exposed workers, we reported a higher bilirubin level due to the exposure [27] [33]. Such results may be a result of the ability of lead to induce eryptosis [34] and the activity of the inducible form of heme oxygenase [35]. Besides, increased bilirubin level may be observed due to the lead hepatotoxicity. Damage to hepatocytes may impair bilirubin conjugation and block biliary tract leading to a release of unconjugated bilirubin into the bloodstream [36]. On the other hand, elevated serum bilirubin level is believed to be able to mitigate lead toxicity thanks to its antioxidant properties. An experimental study showed that bilirubin administration resulted in increased GSH level, enhanced the activity of antioxidant enzymes, and decreased the toxicity of δ-aminolevulinic acid (ALA) in rats [37]. In light of this, lower bilirubin level in the L-Mg group than in the H-Mg group should be regarded as not eligible. The associations between magnesium and bilirubin levels are not well-known. Sarici et al. [38] found a positive association between serum magnesium and bilirubin level in infants with hyperbilirubinemia and postulated magnesium efflux from cells to plasma as a result of the cellular injury related to bilirubin toxicity. In the present study, we also reported a positive correlation between magnesium and bilirubin levels; however, mechanisms underlying this observation need further investigation.

## Conclusions

Low serum magnesium levels contribute to lead-induced oxidative stress, result in unfavorable modification of antioxidant system function, and promote lead-induced impairment of heme synthesis. Besides, serum magnesium level seems to be able to modify the associations between blood lead level and PLT count. Obtained results indicate that prevention of hypomagnesemia should be regarded as an important step in ensuring adequate prophylaxis of chronic lead poisoning.
